# Financial access to health care in Karuzi, Burundi: a household-survey based performance evaluation

**DOI:** 10.1186/1475-9276-8-36

**Published:** 2009-10-24

**Authors:** Sophie Lambert-Evans, Frederique Ponsar, Tony Reid, Catherine Bachy, Michel Van Herp, Mit Philips

**Affiliations:** 1 Médecins Sans Frontières (Belgium), 94 rue Dupré, Brussels, Belgium

## Abstract

**Background:**

In 2003, Médecins Sans Frontières, the provincial government, and the provincial health authority began a community project to guarantee financial access to primary health care in Karuzi province, Burundi. The project used a community-based assessment to provide exemption cards for indigent households and a reduced flat fee for consultations for all other households.

**Methods:**

An evaluation was carried out in 2005 to assess the impact of this project. Primary data collection was through a cross-sectional household survey of the catchment areas of 10 public health centres. A questionnaire was used to determine the accuracy of the community-identification method, households' access to health care, and costs of care. Household socioeconomic status was determined by reported expenditures and access to land.

**Results:**

Financial access to care at the nearest health centre was ensured for 70% of the population. Of the remaining 30%, half experienced financial barriers to access and the other half chose alternative sites of care. The community-based assessment increased the number of people of the population who qualified for fee exemptions to 8.6% but many people who met the indigent criteria did not receive a card. Eighty-eight percent of the population lived under the poverty threshold. Referring to the last sickness episode, 87% of households reported having no money available and 25% risked further impoverishment because of healthcare costs even with the financial support system in place.

**Conclusion:**

The flat fee policy was found to reduce cost barriers for some households but, given the generalized poverty in the area, the fee still posed a significant financial burden. This report showed the limits of a programme of fee exemption for indigent households and a flat fee for others in a context of widespread poverty.

## Background

Although the political situation in Burundi has now stabilised, the civil conflict of 1993-2003 deeply affected the country's inhabitants and infrastructure, particularly the health care system. In order to help rebuild the system, a countrywide cost recovery was implemented in 2002 along with a national policy where the communal authorities were supposed to issue exemption certificates for the poorest who could not afford health care costs [1]. Despite this plan, significant financial barriers to access health care continued to exist. A Save the Children report indicated that "There are serious concerns about the effectiveness of this scheme in protecting the most poor from the cost of illness and in improving their access to health services" [2]. A Médecins Sans Frontières (MSF) survey found that almost a million persons were excluded from health care mainly because of financial reasons and only 0.6% had an exemption certificate [3]. Despite this evidence, the health authorities continued to implement the national cost-recovery policy like many low-income countries which still rely on user fees [4].

Given this situation MSF offered to support health authorities in Karuzi province to develop a project to reduce financial barriers to seeking care in government health facilities. MSF's initial proposal of free care was not accepted by the authorities. Consequently, the project combined reduced fees for everyone and a community-based identification exemption system for indigents. The literature shows evidence that fees impact negatively on care utilisation [5,6] and risks pushing people into poverty. James sees fees as a regressive health financing mechanism [7]. The experience of abolishing user fees on utilisation of health services in South Africa or in Uganda showed positive impact [7-9].

Most documented experiences of maintaining user fees whilst trying to mitigate their impact on the poor through individual-targeting waiver schemes performed weakly [10]. However isolated experiences of subsidies to the poor reached up to 40% of the population through health equity funds in Asia [10] and there are examples in the literature of successful experiences in Latin America [11]. Some authors therefore concluded that the weak performance of many waiver schemes stems from poor policy design and underfunding of the projects [10,12].

In our project, MSF subsidised the cost of care through a combination of reduced flat fee for everyone and free care for indigents identified at community level. The exemption system for indigents incorporated previous experience and recommendations, both in terms of design and funding, in an attempt to overcome weak performance of such schemes in other contexts. The exemption system therefore incorporated previous experience and recommendations [12]: correct and timely financing to compensate structures for lost revenue, proper information to the beneficiaries, pre-identification (or active identification) of households in the community, as well as having an international NGO (MSF) playing the role of driving agent and financier.

### Objective

This article describes the impact on financial access to health care in Karuzi province, Burundi, of a program using (a) a reduced flat fee for any medical consultation and (b) a community-based assessment to exempt the poorest people from any payment, two years after implementation of the system.

## Methods

### Setting

Located in Northeast Burundi, Karuzi was a hilly province [13] with 329,431 inhabitants in 2005 [14]. The population was almost all living in a subsistence economy. There were one referral hospital and 13 health centres (HCs), of which three were private, and 10 were government supported, scattered across the province's seven communes.

### MSF project

MSF had been active in this province since 1993. In 2003, MSF began the community project in cooperation with the provincial government and provincial health authority (BPS) to guarantee financial access to quality health care for Karuzi's population through the support of 10 government HCs and the hospital. MSF involvement included provision of drugs and medical materials, reinforcement of HC management and community involvement, supervision, training and monthly financial incentives for the staff and financial support, as described below. These measures were meant to improve both financial accessibility and quality of care to the population.

There were two financial features of the MSF plan that, when combined, were unique when compared to other similar projects. First, for the large majority of the population, an all-inclusive flat fee of 300 BIF (0.28 US$ in August 2005) per visit was applied for whatever care or treatment was received in all public facilities. The amount of 300 BIF was chosen in collaboration with the BPS based on the income of a daily worker in the fields. By January 2003, the flat fee was implemented for the majority of the population, besides indigents, in all the supported facilities. Fees collected by HCs were used to cover part of their operating costs. MSF compensated HCs for lost fee revenue due to this program.

In the same year, communities across Karuzi elected voluntary members of Health Committees (Hcoms) that were broadly representative of the province's demographics (age, gender, socio-economic status, ethnic group). Hcoms were in charge of disseminating information about the program to the community, ensuring correct implementation of the tariff by the health staff and monitoring problems during the project.

The second financial feature was that the poorest households in Karuzi province were identified by a community level assessment to receive an exemption card entitling them to receive free health care in the supported HCs. This exemption system took into account previous experiences and recommendations in different health contexts, such as making sure there was adequate financial compensation for lost (fee) revenues for the HCs, proper information given to the public about the program, and active identification of indigent households in the community [12].

The exemption system was supposed to ensure that identified households (cardholders) would have access to free health care. However, there were concerns that local health authorities, acting alone, would select a limited number of households because of conflicting interests. This conflict in health service objectives between equity and resource generation has been documented in the literature [15,16]. MSF, therefore, wanted an independent group close to the community to decide on exemptions. Since there was no such existing group, as has been described in other contexts like Cambodia [17], MSF asked the Hcoms to make the assessment, as their members had been elected by and were known by the population of the hills. This targeting mechanism has been advocated in the literature on the basis that more and better quality information is available to communities about their members' resources, needs and circumstances [16].

### Indigent household identification

The Hcoms were charged with identifying indigent households in the communities through socio-economic criteria. These criteria were developed by other Non Governmental Organizations working in the province, together with the provincial authorities (Appendix 1) on the basis of previous assessments [18]. Households meeting at least one indigence criterion were included as beneficiaries. In almost all households, cash availability was low or non-existent so that household income could not be used as an economic criterion as in Cambodia [17] or Thailand [19]. We therefore used the proxy indicators of socio-economic status and vulnerability, such as ownership of land and age and sex of head of household, to capture different aspects of deprivation [16].

Hcom members received training in the community identification procedures by MSF and BPS members. Once they had identified households meeting indigent criteria, random verification by the BPS and MSF was used to avoid nepotism; 10% of all identified households were visited to check their status. The communities also participated in the verification process: MSF and BPS introduced the lists of identified households to meetings with the population where the people could confirm or reject a household's indigent status. Because so many people were poor, stigma attached to receiving a card was low. On the contrary, people hoped to get the cards and avoid financial stress.

Two full-time MSF employees were responsible for listing the identified households, filling out the exemption cards by hand, and organising the administrative system. Exemption cards included information on name, age, sex and "address" of all household members. Hcoms distributed these exemption cards to the identified households. Lists were to be officially updated by Hcoms every two months to add or remove households who had recently become indigent or had improved their situation and no longer qualified. In the end 15% of households (11,247 households) were identified as indigent in early 2005.

### Design

The study included the following MSF data sources: a cross-sectional household survey of the population living in the catchment's area of the 10 public HCs (within 5 km) to determine the financial access to heath care (A), and MSF reports from January 2003 to September 2005 (B).

#### A. Household survey

Karuzi province had 215,470 inhabitants living in the catchment's area of the 10 HCs supported by MSF [13]. Provinces in Burundi were administratively divided into communes, then further divided into hills and under-hills. People did not live in villages but were scattered amongst the hills and the distance from one house to another could sometimes mean 30 minutes walking.

A three-stage, cluster-sampling survey was carried out between 22 August, 2005 and 14 September, 2005. Population data used for the sample was based on the official registration for each commune in the province in 2000. In the first stage of sampling, population figures were used to determine the location of the clusters in the hills. Surveyed hills were randomly selected, taking into account the relative proportion of their population. We used a random number generator to select hills from administration lists. Because some hills were geographically large, "under-hills" in the selected hills were also selected randomly (second stage of sampling).

In the third stage of sampling, within each of the chosen under-hills, the first household to be surveyed was determined by spinning a bottle in the middle of the under-hill to choose a direction. Interviewers then walked until the limit of the under-hill and counted the number of houses in that direction. A table of random numbers was used to choose the first house to visit. After obtaining consent, the head of that household was interviewed. Subsequent questionnaires were successively administered to the households located directly to the right of the front door of the preceding house until the outer edge of the cluster was reached. In case a household was absent or in case the head of household was not available, teams were instructed to revisit the house later in the day. In case they were still absent or unavailable, the household was replaced by the nearest household.

A household was defined as a group of people who shared the same food and slept under the same roof for at least three nights per week.

##### Measurement

The questionnaire included 27 items on the following subjects:

- Household composition.

- Household socio-economic indicators: expenses, access to and use of land.

- Possession of an indigent card and indigence criteria met by the household.

- Health-seeking behaviour for the last episode of illness in the household: whether or not a consultation had been sought, place of consultation or reason for non-consultation and place and completeness of medication received.

- Costs of care and source of the money spent for health care costs.

This study was carried out at the same time as a mortality survey whose recall period, based on previous experience and literature, was three months [20]. For the question related to the episodes of illness, the same recall period was chosen- covering the time from the date of the communal election (3 June, 2005- an important reference in the recent history of the country) up to the date the questionnaire was administered (mean recall period = 90.7 days). Households were asked to relate the most recent episode of illness that occurred during this period. There were no unusual events or changes in the environment such as epidemics or problems at the HCs such as major drugs out of stock or strikes by the health staff during this three-month period.

For this study, we defined having "financial access to primary health care" when a person attended the nearest health centre for a consultation and received a full course of medication on site. Note that in this context virtually everyone was prescribed some medication if they attended a HC. Medication given was then described as full or partial based on the declaration of the respondent. In this study, utilisation of health services at a HC near the house of the respondents was used as a proxy indicator for access. In this context, availability of health structures was not an issue as the sampled households lived around HCs supported by MSF. The choice of the sample of people living at a maximum distance of five km from the HC was made to be sure we would get information about other factors influencing health seeking behaviour besides distance. It is clear however, that for the households living further away, distance would be an additional obstacle.

For people who did not attend the nearest health centre, reasons for their alternative choices or for staying at home were recorded. We categorized barriers to use of health services as financial and non-financial. Households were considered as excluded from health care when they did not seek any care although they felt it was necessary.

When there were no episodes of sickness in the household during the recall period, only questions related to household composition and socio-economic situation were asked.

We used expenses as an indicator of socioeconomic status of the households in the analysis. They were a more stable estimate than income in this type of rural economy, where most people were engaged in subsistence farming, and incomes were influenced by season, and were often irregular or under-estimated [21]. Expenses were measured on the basis of consumption in the household the week before the interview.

Additional information about the households' socioeconomic situation was obtained by using "access to a piece of land" to grow crops as an indicator. The households were divided into three groups: people who did not have access to land, people who owned or rented land for subsistence farming, and people who owned or rented land for farming for profit.

Origin of the money covering the cost of care fell into two main categories: households that had money available (savings, business income) for the costs of care at the time of illness and households that had to mobilise the money. For the latter, we considered that a household was:

- in a "precarious situation" if, in order to pay the cost of care, it had to sell a part of its harvest, which normally fed its members, or if someone in the household had to do extra work.

- "impoverished" if the household had to borrow money or if land, animals or part of a future harvest were sold to pay for the consultation.

The questionnaire was translated into Kirundi, back translated into French and pilot-tested with 30 households in July 2005 by the coordinator of the survey

##### Human resources

Considering the difficulty of carrying out a survey in such a place, eighteen interviewers and three supervisors were recruited. They were selected on the basis of their education level, their knowledge of the province, their ability to speak French and Kirundi fluently and their physical condition. The team received three days of training on survey methods and procedures. Their understanding of the procedures was evaluated through a half-day test of the questionnaire in hills not selected in the sample. The interviewers were regularly monitored by three supervisors headed by a general coordinator during the survey.

##### Sample size

The sample size was calculated using an estimate that 75% of households would have access to primary health care. If the margin of error was fixed at +/- 4% with a cluster effect estimated at 2, then 900 households with at least one sick person in the recall period were required. The calculation used the standard formula: (1.96^2 ^× 2 × (0.75 × 0.25/(0.04)^2^) = 900 [22]. Thirty clusters of 30 households with at least one sick person were visited. However, if a household had no sick person in the recall period, another household was added in order to reach our desired sample size.

##### Data handling

The data was entered daily by two interviewers/encoders into Epi Info 6.04 and checked by the general coordinator and, if necessary, with interviewers and supervisors. The epidemiological/statistical analysis was carried out in Brussels. Geometric means were calculated for costs and expenses. All confidence intervals were calculated at 95% (95%CI). Proportions were compared by the standard chi-squared tests.

##### Ethics

Permissions to carry out the studies were obtained by the BPS and the Provincial authority of Karuzi and approved by the Ethics Review Board of MSF.

#### B. MSF program reports

A document review of MSF materials included the monthly reports of the project, monthly HC data and reports of the expatriates working on the health and management committees from January 2003 to September 2005. Those documents complemented the survey data and were useful for describing the setting, the evolution of the project and the resources needed for the exemption system.

We used the documents as supporting qualitative information about context and programme evolution. We did not undertake a formal qualitative analysis.

## Results

### Description of the sample

A total of 1031 households were visited, 94 households were absent and 937 households were interviewed, representing 4,949 persons. No household refused to be interviewed. The average size of the household was 5.3 persons (95%CI: 5.1-5.5). The age distribution of the sample was similar to national figure for Burundi [23].

Expenses per person were, on average, 375.0 BIF (335.0-420.7) per week.

There were 86.9% (83.8-89.9) of the households who owned a piece of land and 6.4% (3.9-8.9) rented it to provide subsistence farming for their members. Another 1.3% (0.3-2.3) owned/rented a land for profit and 5.4% (3.3-7.6) of households had no access to land at all.

### Results of the exemption system

#### Characteristics of cardholders

Eighty-one households - 8.6% (6.3-11.0)- had an exemption card.

Cardholders had lower weekly expenses and fewer owned a piece of land than non-cardholders (Table 1).

**Table 1:** Comparison between non-cardholders and cardholders

	**Households without cards** (n = 855*)			**Households with cards** **(n = 81)**		
	
	**Amount (BIF)**		**CI 95%**	**Amount (BIF)**		**CI 95%**
**Expenses/pers./week**	**397.2**		**352.4-446.7**	**208**		**133.7-324.3**
	**n**	**%**	**CI 95%**	**n**	**%**	**CI 95%**
Own/rent land for profit	11	1.3%	0.3-2.2	1	1.2%	0.0-3.6
Own land for survival	760	88.90%	85.8-92.0	53	65.40%	53.4-77.5
Rent land for survival	56	6.60%	3.9-9.2	4	4.90%	0.5-9.4
Without land	28	**3.30%**	1.3-5.3	23	**28.40%**	16.7-40.1

* 1 missing

#### Accuracy of the community identification method

Overall, 9.2% (6.1-12.3) of households (86) were found to have indigence criteria as defined in Appendix 1. Thirty-three of them (38%) were landless. As table 2 shows, 44.2% of the households having at least one indigence criterion were cardholders. However, more than half of those with indigence criteria did not have a card (coverage) and almost half of those having a card did not fulfil the criteria (leakage).

**Table 2:** Distribution of cardholders/positive indigence criteria

	**Households with card**			**Households without card**		
	**n**	**%**	**CI 95%**	**n**	**%**	**CI 95%**
	
With indigence criteria	38	44.20%	26.4-62.0	48	55.80%	38.0-73.6
Without indigence criteria	43	5.10%	3.7-6.4	808	95.00%	93.6-96.3

### Access to health care

**Figure 1:**
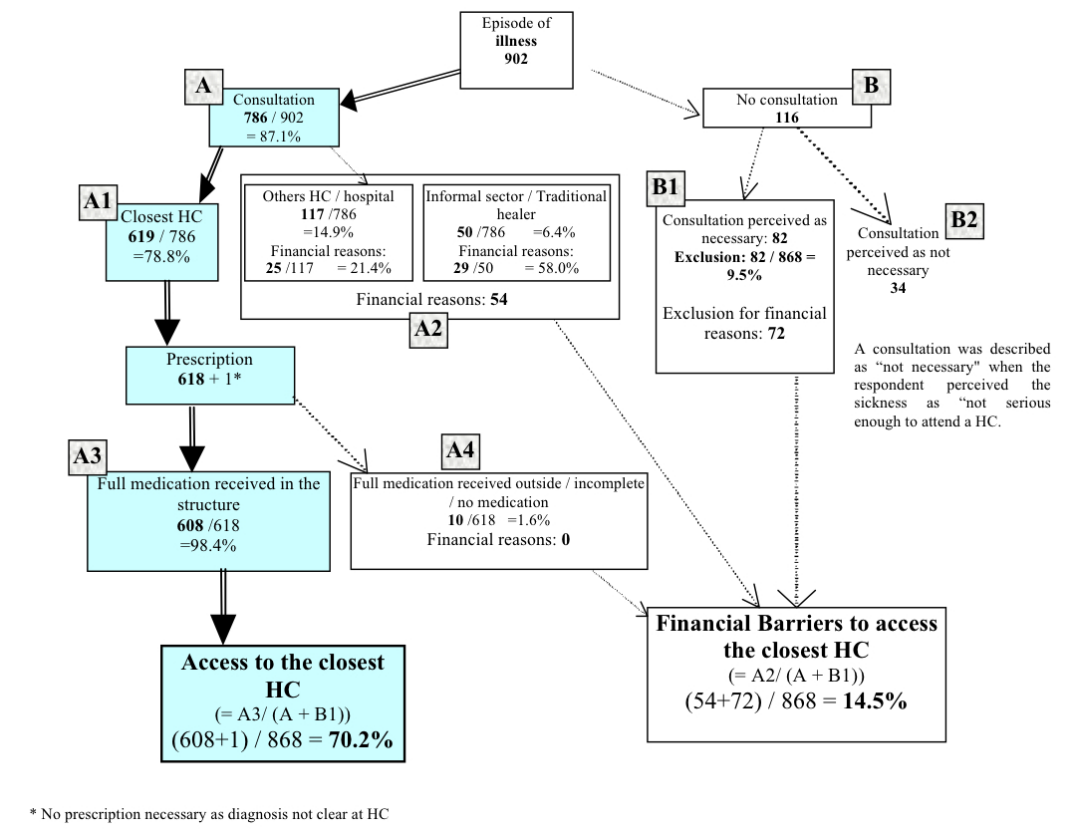
**Access to primary health care**.

In the 937 households surveyed, 902 cases of illness were reported (96.3%). As seen in Figure 1, 87.1% (84.5-89.8) went for a consultation in various locations:

▪ 78.8% (73.4-84.1) sought medical help at the closest HC (supported by MSF applying the flat fee/cards exemption)

▪ 12.1% (6.7-17.5) went to another HC and 2.8% (0.5-5.1) went directly to the hospital. Of these, 21.4% (2.9-39.8) explained their choice was due to financial barriers. Other barriers included a lack of confidence with the HC staff or a shortage of medication at the HC.

▪ 6.4% (4.5-8.2) went to the informal sector/traditional healers, 58.0% (45.2-70.8) claimed to do so for financial reasons.

Of those who consulted in the closest HC, 98.4% (97.3-99.4) received full treatment.

In total, 70.2% (64.9-75.5) of the population had access to care at their local HC.

However, 9.5% (7.0-12.0) of the households were excluded from health care and for 87.8% of them (76.1-94.5); this was due to financial barriers. Overall, 14.5% (10.5-18.5) of households experienced financial barriers that prevented them from accessing care at their closest HC.

#### Access to health care for cardholders

Of the cardholders, 91.1% (72/79) went for a consultation for the last episode of sickness and 88.9% of these (64/72) went to the closest HC. Of those going to the closest HC, 96.9% (62/64) obtained full treatment but four of the cardholders paid 300 BIF. Access to health care among cardholders was higher than non-cardholders; 80.5% (CI 95%: 72.4-88.6) vs. 69.2% (CI 95%: 63.3-75.0). Although this difference did not reach statistical significance, the relative risk was significant; cardholders had 1.58 (0.99-2.54) more chance to access health care than non-cardholders.

### Costs of care

Of the people who went for a consultation at the closest HC (n = 619),

- 12.0% (8.8-15.1) did not pay for health care (74 cases) and 81.1% (71.8-98.3) had a card (60 cases).

For those who paid,

- The average amount paid was 311.2 BIF (304.8-317.7) or median amount of 300 BIF.

- 6.3% (1.8-15.7) of the cardholders (4 cases) still paid the flat fee,

- 2.5% (1.2-3.8) of non-cardholders (14 cases) did not pay for health care.

#### Origin of the money and effects of the cost of care on households

Of the cases who went for a consultation at the closest HC, Table 3 shows that only 13.1% of the households had the money available at the moment of the disease.

**Table 3:** Situation of households that paid for health care

	n = 544*	**%**	**CI 95%**
	
Money available	71	13.10%	9.0-17.1
Precarious	332	61.00%	54.3-67.8
Impoverished	141	25.90%	20.3-31.6

* 1 missing

Of the rest, 61.0% entered into a precarious situation because they had to look for extra work outside of the household (123 cases) or they had to sell a part of their harvest that they would normally have used for their own food (209 cases) and 25.9% of the households became impoverished. Most of them went into debt (116 cases), some had to sell part of their future harvest (20 cases) and a few sold animals or land (5 cases).

## Discussion

This study showed that despite an innovative program that combined a flat fee for care for most people with an exemption system for indigents, there continued to be significant financial barriers in accessing health care in Karuzi. Even though access to health care was readily available for 70% of overall population, about 30% sought care elsewhere or stayed home. Why was this so?

### 1. Significant overall poverty

A key feature was that the national poverty line in rural areas was evaluated at 1031.73 BIF/person/week [24], so that 88.0% (83.5-91.6) of the population in Karuzi lived below that threshold at the time of the survey, confirming a context of widespread generalized poverty. Cash available at the household level was limited and it was difficult to mobilise quickly in the face of unexpected expenses for an episode of illness.

Although most households in the province owned a piece of land, it was just enough for subsistence, not for profit. Assessments carried out at the national level revealed that the average size of land per household was less than 0.5 ha [25], barely enough to feed household members, let alone generate income. The situation became worse during the crisis that affected the country during the past decade when many households had to sell their land [25]. "Landless" was the main indigence criterion reported in the survey.

### 2. The exemption system: mixed results

The exemption system was found to protect only 8.6% (6.3-11.0) of the population. This was lower than the 15% of households identified by the Hcoms but larger than in other provinces in Burundi [3] or in other countries where classical exemption mechanisms were in place [26-28]. Exemption schemes, even those endorsed by policy and legislation, are rarely fully effective. Schemes aimed at targeting the poor with exemptions often miss the intended beneficiaries, those in greatest need [29].

MSF studies have shown that classical exemption mechanisms in Mali, Sierra Leone or Haiti covered less than 2% of the population" [30]. And Ridde [31] noticed that, although exemption of payment for indigents was one of the core principles of the Bamako Initiative, in many settings, exemptions had not been implemented or had not been able to protect very many people in the community.

However, results similar to those of our study were found with equity funds in Cambodia [29,32]. Their design was close to the exemption system developed in Karuzi and included the existence of donor funding, the presence of a driving agent, clear separation of roles, and appropriate identification techniques. These studies reached conclusions similar to ours: while these mechanisms are superior to traditional waiver systems in terms of health services utilisation by the target group, studies reveal remaining barriers to access and indebtedness prevention.

However, existing studies tend to focus on hospital and health structure data while failing to provide information on non-users of the system. Our study therefore brings additional data on general population access. These data are crucial to be considered for national policy choices.

Our results showed that cardholders had better financial access to health care than non-cardholders. It was also encouraging that almost all cardholders benefited from free health care in public HCs.

Although most of the cardholders were in a worse socio-economic situation than the rest of the population, the exemption system had problems properly covering the poor as shown by 56% of the households who had at least one indigent criterion but did not get cards. One reason for this low coverage could be the time lag between the date of identification and the survey. This would reinforce the idea that exemption systems do not capture properly the dynamic dimension of poverty. At the same time, 5% of households did get cards despite not having met the criteria. As well, although Hcoms had identified 15% of households as indigent, our survey found only 8.6% of households in possession of a card. This difference could be explained by the following:

▪ Around 400 cards (3.5% of all the cards distributed) were never distributed to the identified households because the householders were absent during the distribution meetings and the Hcoms did not follow up.

▪ Initially, some health staff members were reluctant to accept the exemption system and confiscated cards when they considered that beneficiaries did not qualify as indigent. This situation improved with time thanks to the reporting of the Hcoms.

▪ Cards lost were often not renewed by the Hcoms.

These difficulties should have been handled by the Hcoms with updates to the list every two months. However, this would have implied constant and time-consuming re-assessment. Experience in this project showed that maintaining an accurate list was a complicated and imperfect process, very demanding both financially and in terms of human resources. Members of Hcoms were not paid and this may have affected their motivation.

Furthermore, the performance of the system was hampered by the difficulty in defining and interpreting indigence criteria in a context of generalised poverty. The feasibility and accuracy of distinguishing the poor from the non-poor to determine eligibility for exemptions, is fraught with problems [28,33]. The notion of indigence is complex and covers both poverty and social exclusion [34]. Criteria might have been subjectively and arbitrarily interpreted even though the members of the health committees had been trained to recognise eligible households. "A major difficulty is to identify very poor people in a population in which the poverty is rife" [33].

The exemption system was demanding in terms of other human and financial resources. In addition to the Hcom members' time, human resource inputs included significant time of two full-time staff members, financed by MSF. Significant financial costs arose from MSF's financial support to HCs for compensation of revenue lost due to exemptions, considered a key condition for fee exemption schemes to be pro-poor [12]. This raises the question of whether such resources should be provided to a system which benefits a relatively small number of people, particularly in a context of poverty. Other authors argued that "A universal free healthcare approach is justified in all situations with widespread misery or when time does not allow individual assessment schemes to be implemented [...]. Alternatively, identifying people living in poverty (by proxy means testing) and targeting benefits to them could be more attractive than a universal approach if the proportion of poor people in the society is not overwhelming..." [10]

### 3. Access to health care

Despite these difficulties there were some positive elements to the project. In 2003, for all of Burundi, the level of access to health care in areas where cost-recovery was implemented was 58% of the population [3]. In the Karuzi project, access to health care was much better, as 70% of the population went for a consultation to the closest HC and received full medication. Other encouraging results were that consultations in the informal sector were few (6.4%) as compared to other African contexts [30].

Although mechanisms set up in the project to improve access proved to have done so compared to other areas in Burundi where cost recovery schemes were implemented, they still revealed important limitations in their potential to increase the use of health services for the population. Almost 15% still had financial barriers to access the closest HC and 10% of the population remained excluded from health care mainly for financial reasons. These findings were in line with other surveys revealing that even low fees constituted an obstacle to patients' access in contexts of widespread poverty [30,35]. MSF's experience has also revealed that targeting strategies compared poorly to general exemption or those based on large categories (like women and children), such as those implemented in Burundi in 2006 (national free care policy for under-5s and pregnant women). In addition, in contexts where health fees were totally abolished for all patients, evidence has shown an increase in the use of health services and specific benefits for the poorest households [35].

### 4. Cost of care, financial burden in households

A specific feature of the project was the implementation of the reduced flat fee system. Although the amount of the agreed fee seemed minimal and was respected by the staff, one visit to the primary health care level represented almost the equivalent of one day of household expenses. Eighty-seven percent of households reported not having sufficient money available to seek immediate care for their last illness episode. To finance health care, more than a quarter became impoverished and 60% needed extra work to provide cash flow, which risked delaying consultation. Thus, despite the reduced flat fee, the cost of health care still represented an important burden for many households. Further, our study did not include indirect costs linked to treatment such as transportation, food expenditure and loss of time [35]. These costs can initiate a vicious circle underlined by Noirhomme et al. [32] in which "poverty not only brings ill-health, but ill-health also tends to worsen poverty".

### Limitations

There were a number of limitations to the study. MSF was well known in the province especially for the identification of indigents. Although the surveyors - MSF employees - clarified that they were not in charge of identifying indigents in the community, respondents might have answered questions in a way to maximise their benefit, for instance, by overestimating their expenses in hope of being included on the indigent lists. This factor is likely to have been limited given the very low level of expenses reported. If present, this bias would lead to an underestimation of real poverty levels rather than an overestimation, as expenses were used to assess the socio economic status of households. As well, confronted by a western medical organisation, the respondents might have underreported the use of traditional medicines or of the informal sector.

An additional concern may be the 90.7-day recall period for last illness episode, which may have reduced the accuracy of details recalled. However, potential recall bias was reduced since the illness of inquiry was the most recent during the recall period.

## Conclusion

An innovative approach of adopting a flat fee for consultations in primary care clinics in rural Burundi and identifying indigents was somewhat successful in increasing financial access to care. However, against a background of widespread poverty, and the difficulties to properly target the poor in such a context, many people still did not obtain appropriate care or suffered financial hardship doing so. Introducing these measures was cumbersome, and not really responsive to the poverty dynamics in the population. These results indicate that alternative strategies, such as free care for everyone or targeted groups such as under-5s or pregnant women as implemented in Burundi in 2006, are needed to ensure increased access to effective health services for the poor.

## Competing interests

The authors declare that they have no competing interests.

## Authors' contributions

SLE, FP, CB, MVH, TR, and MP contributed to study conception and design, interpretation of the data, and drafting and revising the manuscript; SLE and FP led data collection; CB led the statistical analysis with additional analysis by SLE and MVH. All authors read and approved the final manuscript.

## Appendix 1 - Exemption criteria

### Groups identified by Hcom

° Long-term welfare recipient: Socially isolated, without children, often elderly without family. No income or savings; inadequate food, clothes and accommodation. Lives with the support of the neighbourhood. Often disabled and/or with serious health problems.

° Without land/property: live in hut, seasonal workers.

° Disabled people in household with no member capable of working, not receiving assistance from outside the household.

° Elderly (over 55 years old) in household with no member capable of working, not receiving assistance from outside the household.

° Orphans head of household (below 18 years old) not receiving assistance from outside the household.

° Foster care and orphans care, taking care of indigents, in household with no member capable of working, not receiving assistance from outside the household.

° Widowed, head of household, in a household with no member capable of working; low income; not receiving assistance from outside the household.

### Groups identified by HCR, directors of school

° Repatriated households, 6 months after their return.

° Students meeting above criteria.

## References

[B1] République du Burundi. Ministère de la Santé publique et Ministère de l'intérieur et de la sécurité publique (2003). Ordonnance ministérielle n° 630630/445 du 02/04/2003 portant fixation des modalités de prise en charge médico-sanitaire des indigents Bujumbura.

[B2] Save the Children UK (2003). An Unnecessary Evil? User Fees for Healthcare in low-income Countries The Cost of Coping with Illness Burundi Briefing London.

[B3] MSF-Belgium (2004). Access to healthcare in Burundi: results of three epidemiological surveys Research and analysis report Brussels.

[B4] World Health Organisation (2008). The World Health report 2008 Primary Healthcare Now ore than Ever Geneva.

[B5] Creese AL (1991). User charges for health care: a review of recent experience. Health Policy Plan.

[B6] Palmer N, Mueller DH, Gilson L, Mills A, Haines A (2004). Health financing to promote access in low income settings - how much do we know?. Lancet.

[B7] James C, Hanson K, Mcpake B, Balabanova D, Gwatkin D, Hopwood I, Kirunga C, Knippenberg R, Meessen B, Morris SS, Preker A, Souteyrand Y, Tibouti A, Villeneuve P, Xu K (2006). To retain or to remove user fees? Reflections in the current debate in low and middle income countries. Applied Health Econ Health Policy.

[B8] Wilkinson D, Gouws E, Sach M, Karim SS (2001). Effect of removing user fees on attendance for curative and preventive primary health care services in rural south Africa. Bull World Health Organ.

[B9] Nabyonga J, Desmet M, Karagami H, Kadama PY, Omaswa FG, Walker O (2005). Abolition of cost sharing is pro-poor: evidence from Uganda. Health Policy Plan.

[B10] Sepheri A, Chermonas R (2001). Are user charges efficiency and equity-enhancing? A critical review of economic literature with particular reference to experience from developing countries. J Int Dev.

[B11] Gwatkin DR (2000). The current state of knowledge about Targeting health programmes to reach the poor.

[B12] Bitràn R, Giedion U (2003). Waivers and Exemptions for Health Services in Developing Country. Social Protection Discussion Paper Series.

[B13] Protopopoff N, Van Herp M, Maes P, Reid T, Baza D, D'Alessandro U, Van Bortel W, Coosemans M (2007). Vector control in a malaria epidemic occurring within a complex emergency situation in Burundi: a case study. Malar J.

[B14] Protopopoff N, Van Bortel W, Marcotty T, Van Herp M, Maes P, Baza D, D'Alessandro U, Coosemans M (2007). Spatial targeted vector control in the highlands of Burundi and its impact on malaria transmission. Malar J.

[B15] Hardeman W, Van Damme W, Van Pelt M, Por I, Kimvan H, Meessen B (2004). Access to health care for all? User fees plus a Health Equity Fund in Sotnikum, Cambodia. Health policy Plan.

[B16] Hanson K, Worrall E, Wiseman V, Mills A, Bennett S, Gilson (2006). Targeting services towards the poor: a review of targeting mechanisms and their effectiveness. Health, economic development and household poverty: from understanding to action.

[B17] Bart J, Neil P (2006). Improving access for the poorest to public sector health services: insights from Kirivong Operational Health District in Cambodia. Health Policy and Plan.

[B18] Africare/Burundi (2001). Mise en oeuvre de la composante "développement communautaire" du PDRDMR en province de Karusi Rapport sur le ciblage de la pauvreté dans la province de Karuzi Washington, Bujumbura.

[B19] Mills A (1991). Exempting the poor: the experience of Thailand. Soc Sci Med.

[B20] Checchi F, Robert L (2005). Interpreting and using mortality data in humanitarian emergencies.

[B21] Dercon S, Clark DA (2006). Poverty measurement. The Elgar Companion to Development Studies.

[B22] Kish L (1965). Survey Sampling.

[B23] République du Burundi. Ministère de la Santé Publique (2005). Plan National de Développement Sanitaire 2006-2010 Bujumbura.

[B24] République du Burundi (2003). Poverty Reduction Strategy Paper Bujumbura.

[B25] Centre d'Alerte et de Prévention des Conflits (CENAP) (2005). Pratiques rurales en matière de gestion des propriétés foncières, rapport final Bujumbura.

[B26] McPake B, Hanson K, Mills A (1992). Experience to Date of Implementing the Bamako Initiative: a review and five Country Case Studies.

[B27] Russell S, Gilson L (1997). User fee policies to promote health services access for the poor: a wolf in sheep's clothing?. Int J Health Serv.

[B28] Gilson L (1997). The lessons of user fee experience in Africa. Health Policy Plan.

[B29] Jacobs B, Price NL, Oeun S (2007). Do exemptions from user fees mean free access to health services? A case study from a rural Cambodian hospital. Trop MedI Int Health.

[B30] MSF-Belgium (2008). No cash, no care: how "user fees" endanger health An MSF briefing paper on financial barriers to Healthcare Brussels.

[B31] Ridde V, Preker AS (2004). L'initiative de Bamako 15 Ans Après. Un Agenda Inachevé. Health, Nutrition and Population Discussion Paper.

[B32] Noirhomme M, Meesen B, Griffiths F, Ir P, Jacobs B, Thor R, Criel B, Van Damme W (2007). Improving access to hospital care for the poor: comparative analysis of four health equity funds in Cambodia. Health Policy Plan.

[B33] Whitehead M, Dahlgren G, Evans T (2001). Equity and health sectors reforms: can low income countries escape the medical poverty trap?. Lancet.

[B34] Kaddar M, Schmidt-Ehry B, Stierl F, Tchicaya A (1997). Indigence et Accès aux Soins de Santé en Afrique Sub-Saharienne: Situation et Perspectives d'action. Eschbron: GTZ.

[B35] Meessen B, Van Damme W, Kirunga Tashobya C, Tibouti A (2006). Poverty and user fees for public health care in low-income countries: lessons from Uganda and Cambodia. Lancet.

